# ExplaineR: an R package to explain machine learning models

**DOI:** 10.1093/bioadv/vbae049

**Published:** 2024-03-26

**Authors:** Ramtin Zargari Marandi

**Affiliations:** Centre of Excellence for Health, Immunity and Infections (CHIP), Rigshospitalet, Copenhagen University Hospital, DK-2100 Copenhagen, Denmark

## Abstract

**Summary:**

SHapley Additive exPlanations (SHAP) is a widely used method for model interpretation. However, its full potential often remains untapped due to the absence of dedicated software tools. In response, *ExplaineR*, an R package to facilitate interpretation of binary classification and regression models based on clustering functionality for SHAP analysis is introduced here. It additionally offers user-interactive elements in visualizations for evaluating model performance, fairness analysis, decision-curve analysis, and a diverse range of SHAP plots. It facilitates in-depth post-prediction analysis of models, enabling users to pinpoint potentially significant patterns in SHAP plots and subsequently trace them back to instances through SHAP clustering. This functionality is particularly valuable for identifying patient subgroups in clinical cohorts, thus enhancing its role as a robust profiling tool. *ExplaineR* empowers users to generate comprehensive reports on machine learning outcomes, ensuring consistent and thorough documentation of model performance and interpretations.

**Availability and implementation:**

*ExplaineR* 1.0.0 is available on GitHub (https://persimune.github.io/explainer/) and CRAN (https://cran.r-project.org/web/packages/explainer/index.html).

## 1 Introduction

Machine learning, especially its application in processing clinical datasets, has gained immense popularity in recent years. As the introduction of new complex algorithms allows the machine learning field to grow, it also comes along with the challenge of unravelling how these algorithms produce an output. For example, tree-based ensemble models (e.g. Random Forests and Gradient Boosting Machines), offer robust predictive capabilities but with limited interpretability (Lou *et al.* 2013, Chen and Guestrin 2016). Existing methods to extract feature importance from such models are subject to limitations, leading to inconsistencies in feature importance (Saarela and Jauhiainen 2021). For example, Gini importance measures how frequently a feature is used to split the data across all trees, and this frequency measurement determines the feature importance. Gini importance however, tends to inflate the importance of continuous or high-cardinality categorical variables, and it can underestimate the importance of correlated features. In XGBoost and LightGBM, Gain importance measures the contribution of a feature to improvement in a model’s performance (e.g. reduction in loss function) when it is used in a split. Gain importance can be biased towards continuous features leading to inaccurate importance values for categorical variables or those with many levels. In contrast to black box models, white box models, like linear regression, are more transparent but often less powerful in handling complex data patterns (James *et al.* 2013).

The critical issue of model interpretability has spurred the development of methods that simplify our understanding of model decisions. SHapley Additive exPlanations (SHAP) analysis, a novel approach grounded in cooperative game theory, has emerged as a prominent tool in this realm (Lundberg and Lee 2017). It quantifies the contribution of each feature to a model’s prediction, thereby enhancing the interpretability of complex models (Zucco *et al.* 2022).

Although model performance measures (e.g. Matthews correlation coefficient in binary classification) are essential for evaluating models, their integration in SHAP summary plots is often neglected. These plots typically represent both accurate and erroneous predictions without distinguishing between them (Chicco and Jurman 2020). Additionally, visual tools such as Decision Curve Analysis are vital for understanding model performance but are sometimes overlooked (Vickers and Elkin 2006). Model fairness, particularly in medical and clinical research, is another critical aspect of model interpretation that requires attention (Obermeyer *et al.* 2019, Suresh and Guttag 2021).

SHAP analysis represents a relatively recent methodology for extracting information regarding the importance of features in both individual samples and groups of samples. Unlike permutation-based methods, SHAP analysis not only provides information on importance but also indicates the direction of feature contribution. However, a notable challenge arises from inconsistent reporting or misinterpretation. For instance, when contributions to model predictions are incorrectly interpreted as causal or direct effects on the outcome.

Furthermore, certain patterns within SHAP summary plots, delineated by graphical homogeneities in the effects of features on model outputs, are disregarded. This is largely due to the visual intricacies arising from an excessive number of visualized samples. Nevertheless, some of the disregarded patterns may contain vital information that is necessary for the characterization of sample clusters that could potentially represent specific phenotypes. This holds particular significance in scenarios where understanding the influence of a feature within a subset of patients is imperative, similar to the sensitivity analysis paradigm prevalent in clinical research.

In response to these challenges, the *ExplaineR* package in R is introduced here to provide a comprehensive framework for interpreting machine learning models primarily focused on binary classification and regression models. This package aims to standardize reporting of model performance and interpretation, facilitating its use across diverse research fields, including medical and clinical sciences.

## 2 Overview

*ExplaineR* package was developed using the R programming language (4.1.3) based on popular and well-documented R packages including *mlr3* (0.14.0) (Lang *et al.* 2019), *CVMS* (1.3.4) (Olsen and Zachariae 2022), and *iml* (0.11.0) (Molnar *et al.* 2018). *ExplaineR* package has been published at the Comprehensive R Archive Network (CRAN). The analytical foundation of the package is based on SHAP analysis (Lundberg and Lee 2017), that is the calculation of SHAP values for each feature to determine their predictive importance. The core functionality of the package lies in the integration of k-means clustering (Hartigan and Wong 1979) in SHAP analysis as well as providing unified access to important yet overlooked model evaluation methods such as fairness analysis and decision curve analysis. All functions of *ExplaineR* package are outlined in Table 1.

**Table 1.** List of functions in the *ExplaineR* package and the availability of similar functions in the *shapr* and *iml* packages in R and the *shap* package in Python.

Function name	Short description	*shapr*	*shap*	*iml*
eCM_plot	Generates a confusion matrix plot incorporating sensitivity, specificity, positive predictive value (PPV), and negative predictive value (NPV) visualizations based on *CVMS* package.	NA^a^	NA	NA
eDecisionCurve	Conducts decision curve analysis, quantifying the net benefit of models across decision thresholds, crucial for assessing clinical utility in medical research.	NA	NA	NA
eFairness	Generates precision–recall and receiver-operating characteristics (ROC) curves to facilitate fairness analysis of a binary classification model, particularly assessing performance across user-defined subgroups.	NA	Available	NA
ePerformance	Constructs ROC and precision–recall curves, including threshold information, offering a comprehensive evaluation of binary classification model performance. It calculates the area under the curve of ROC curve (AUC) and precision–recall curve (PRAUC), balanced accuracy, Matthews correlation coefficient, brier score, PPV, NPV, specificity, and sensitivity.	NA	NA	NA
eROC_plot	Generates precision–recall and ROC curves, essential graphical representations for evaluating the performance of binary classification models.	NA	NA	NA
eSHAP_plot	Estimates Shapley (SHAP) values from cooperative game theory to produce an enhanced SHAP analysis for binary classification models by depicting interactive SHAP summary plots highlighting correct and incorrect predictions and edges between nodes representing each sample.	Available^b^	Available^b^	Available^b^
eSHAP_plot_reg	Estimates SHAP values for regression models.	Available^b^	Available^b^	Available^b^
SHAPclust	Applies k-means clustering to data samples based on SHAP values, revealing subgroups with distinct patterns of feature contributions in a binary classification model as well as highlighting correct and incorrect predictions.	NA	Available^b^	NA
ShapFeaturePlot	Creates an interactive plot illustrating the association between SHAP values and feature values, providing insights into the impact of features on model predictions, and highlighting correct and incorrect predictions.	NA	Available^b^	Available^b^
ShapPartialPlot	Generates an interactive partial dependence plot based on SHAP values, illustrating the marginal effect of features on the predicted outcome of a machine learning model, highlighting correct and incorrect predictions.	NA	Available^b^	Available^b^
regressmdl_eval	Conducts evaluations of a regression model by calculation of mean squared error (MSE), root mean squared error (RMSE), mean absolute error (MAE), and *R*-squared (*R*²).	NA	NA	NA

a NA, not available.

b Similar functions are available in these packages but without the interactive feature for data enquiry and without highlighting correct and incorrect predictions (i.e. classification).

The availability of the functionalities of *ExplaineR* in comparison with three popular packages, namely, *shapr* (Aas *et al.* 2021), *shap* (Lundberg and Lee 2017, Lundberg *et al.* 2020), and *iml* is outlined in Table 1. There are several practical components that distinguish the *ExplaineR* package from other packages: (i) *ExplaineR* indicates which samples correspond to correct or incorrect predictions (in classification tasks), providing users with key information for model interpretation, (ii) the visualizations are interactive, allowing users to pinpoint specific samples to query for additional information, (iii) *ExplaineR*’s SHAP summary plot can depict categorical and missing features, (iv) SHAP clustering in the *ExplaineR* package generates SHAP summary plots for the clusters of samples that can be compared to the (overall) SHAP summary plot where all the samples are depicted, (v) in a SHAP summary plot generated by the *ExplaineR* package, feature ranking is based on correctly classified samples rather than all samples. This distinction is sometimes overlooked, but it helps to differentiate between assessing the impact of features on the model (when the mean of absolute SHAP values is calculated across all samples) and determining the actual importance of features, as confirmed by the ground truth labels.

Of note, *ExplaineR* focuses on binary classification and regression models, whereas for example the *shap* package covers a wider variety of machine learning and deep learning models. There are also some other software solutions like *H2O* (LeDell and Poirier 2020) for model interpretation. A key difference is that *ExplaineR* offers users the flexibility of designing custom ML pipelines while *H2O* is useful for autoML frameworks. Another important difference is that *H2O* explainability does not provide the granular information about SHAP clusters and interactive visualizations that *ExplaineR* provides.

### 2.1 Model interpretation

**Figure 1.**
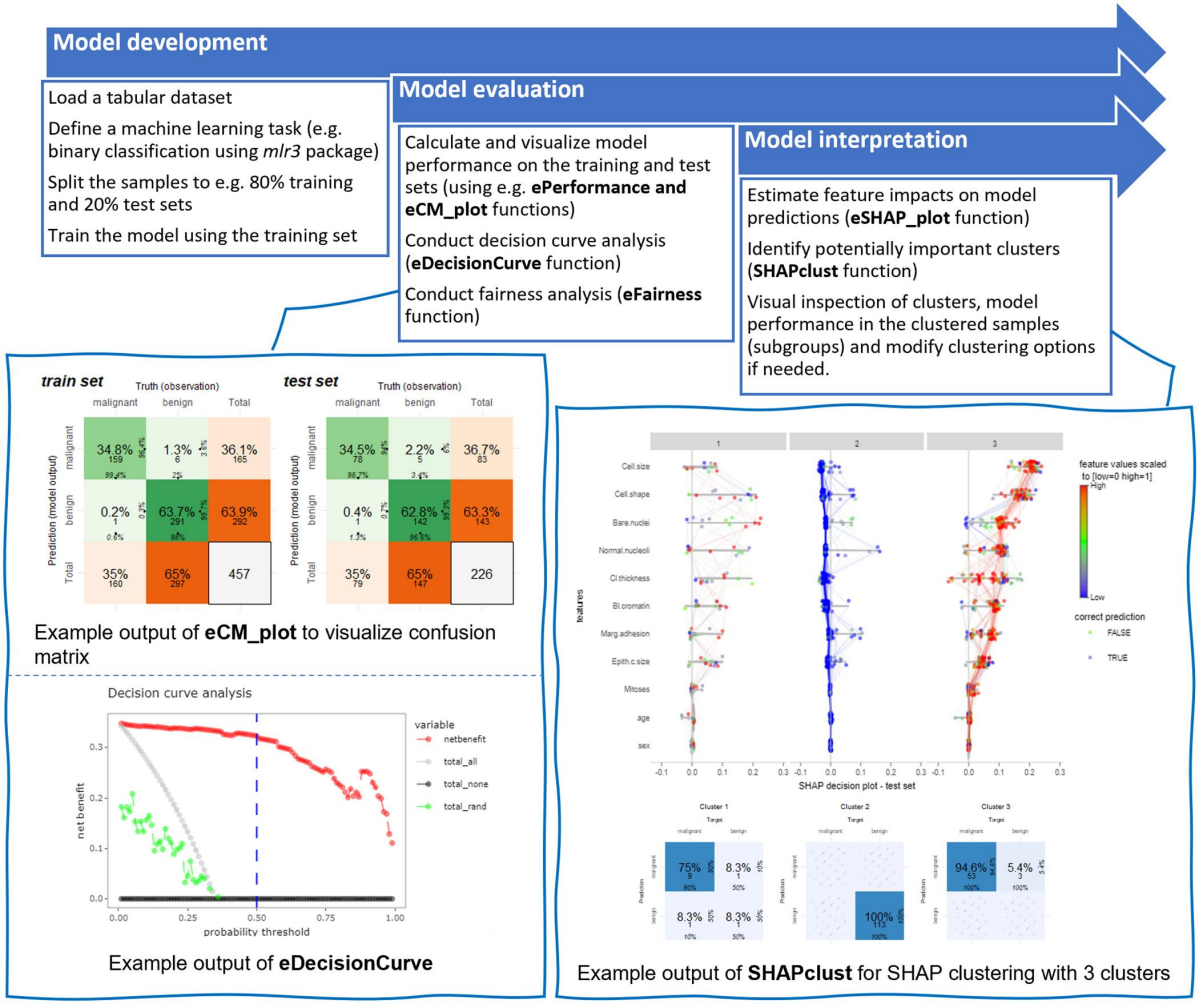
Workflow overview for machine learning analysis using the *ExplaineR* package showcased on a random forest model (binary classification) based on the Wisconsin Breast Cancer dataset. On the left panel, confusion matrices for the training and test sets as well as a plot to compare the model net benefit with alternatives are shown. On the right panel, SHAP summary plots for three clusters of samples resulting from SHAP clustering and their according confusion matrices are displayed. More details are available on the GitHub repository of the package (https://persimune.github.io/explainer/).

A brief overview of the workflow for machine learning analysis beginning from model development to model evaluation and interpretation is described in Fig. 1. SHAP summary plots are usually presented with features sorted on the *y*-axis in descending order by their overall impact as quantified by mean absolute SHAP values across all samples (instances or data points) and SHAP values on *x*-axis. The feature values are encoded by color. K-means clustering is applied on SHAP values and thereby the samples are divided into subgroups according to pattern similarities in the SHAP summary plot. *ExplaineR* generates subplots according to subgroup and allows exporting of information about model performance on those subgroups and their feature values. This is an important capability for data-driven profiling of data samples associated with individuals, e.g. patients (Zargari Marandi *et al.* 2023).

SHAP clustering is valuable in disease outcome prediction based on clinical data. For example, in binary classification tasks, after clustering samples on a test set to three clusters based on SHAP values of features, the clusters are expected to characterize three patient subgroups. One cluster will include the samples with the majority being predicted to have high risk of developing a disease (reflected by higher SHAP values). Similarly, a subgroup of patients with lower risk of developing the disease will be characterized by a second cluster. In practice, models have some samples with weak or uncertain predictions (i.e. predicted probabilities tend toward the chance level). The subgroup of patients from uncertain predictions is expected to be characterized by the third cluster. This can be used for both model and data diagnosis as well as in generalization of the model in which one could predict whether a model would perform well for new patients based on how close their feature vectors are to the known subgroups of patients that were identified by the SHAP clustering method.

SHAP analysis indicates the direction of feature impact in addition to the magnitude of feature importance. This directional information is crucial for understanding whether an increase or decrease in a particular feature value leads to an increase or decrease in model predictions. SHAP values can also reflect nonlinearities and feature interactions subject to the assumption of additivity, meaning that the contribution of each feature to a prediction is considered independently. These advantages and limitations should therefore be considered in model interpretation.

### 2.2 Model evaluation

As an important part of machine learning analysis, the *ExplaineR* package provides computation of multiple measures in tabular format (see Table 1). This allows for general and detailed assessments of model performance that are useful for comparing different models. In addition, the area under receiver operating characteristics curve (AUC-ROC), precision–recall curve with threshold levels, and confusion matrices are visualized with threshold levels and user-interactive options to obtain additional information. In addition, decision curve analysis (Vickers and Elkin 2006) is provided to assess the performance of binary classification models against alternative models or conditions such as random guessing, or extreme cases with all-positives or all-negatives (Fig. 1).

### 2.3 Machine learning in R

*ExplaineR* is a versatile package that can be utilized on diverse tabular datasets. In these datasets, rows typically represent samples (e.g. observations, instances, records, data points), while columns represent features, including an outcome variable. The outcome variable may be binary for binary classification tasks or continuous (numerical) for regression models. The package documentation that is publicly available on GitHub provides tutorials on how to use *ExplaineR* on a modified Breast Cancer Wisconsin dataset to try out all package functionalities for both classification and regression tasks. This sample dataset includes both numerical and categorical features presented in a tabular format.

All models that are already wrapped as *mlr3* learners are supported by *ExplaineR*. Furthermore, most popular models from other packages were successfully run by *ExplaineR* without errors, including random forest (*ranger* package), XGBoost (*xgboost* package), LightGBM (*ligthgbm* package), and imbalanced random forest (*randomForestSRC* package).

*ExplaineR* addresses some of the challenges that arise in the application of SHAP analysis, including inconsistent reporting and misinterpretations. The availability of the package in an R statistical computing environment would encourage researchers who may not have extensive programming experience to apply this analysis as a key part of their machine learning pipelines.

## 3 Conclusion

In conclusion, the *ExplaineR* package provides new analytical features in decoding complex machine learning models. It wraps a suite of practical tools for researchers in various domains, particularly in medical and clinical research. Its implications offer critical insights for model interpretation and evaluation.

## Data Availability

The Breast Cancer Wisconsin is available from *mlbench* package in R.

## Code availability

The source codes for *ExplaineR* package are publicly available at https://github.com/PERSIMUNE/explainer.

## References

[vbae049-B1] Aas K , Jullum M, Løland A. Explaining individual predictions when features are dependent: more accurate approximations to shapley values. Artificial Intelligence 2021;298:103502.

[vbae049-B2] Chen T , Guestrin C. XGBoost: a scalable tree boosting system. In: *Proceedings of the 22nd ACM SIGKDD International Conference on Knowledge Discovery and Data Mining*, New York, NY, USA. Association for Computing Machinery, 2016, 785–94.

[vbae049-B3] Chicco D , Jurman G. The advantages of the Matthews correlation coefficient (MCC) over F1 score and accuracy in binary classification evaluation. BMC Genomics 2020;21:6. 31898477 10.1186/s12864-019-6413-7 PMC6941312

[vbae049-B4] Hartigan JA , Wong MA. Algorithm as 136: a k-means clustering algorithm. J R Stat Soc Ser C Appl Stat 1979;28:100–8.

[vbae049-B5] James G , Witten D, Hastie T, Tibshirani R. An Introduction to Statistical Learning. New York, NY: Springer, 2013.

[vbae049-B6] Lang M , Binder M, Richter J et al mlr3: a modern object-oriented machine learning framework in R. J Open Source Softw 2019;4:1903.

[vbae049-B7] LeDell E , Poirier S. H2O AutoML: scalable automatic machine learning. In: *Proceedings of the AutoML Workshop at ICML,* San Diego, CA, USA. AutoML, 2020.

[vbae049-B8] Lou Y , Caruana R, Gehrke J, Hooker G. Accurate intelligible models with pairwise interactions. In: *Proceedings of the 19th ACM SIGKDD International Conference on Knowledge Discovery and Data Mining.* New York, NY: ACM, 2013, 623–31.

[vbae049-B9] Lundberg SM , Erion G, Chen H et al From local explanations to global understanding with explainable AI for trees. Nat Mach Intell 2020;2:56–67. 32607472 10.1038/s42256-019-0138-9 PMC7326367

[vbae049-B10] Lundberg SM , Lee S-I. A unified approach to interpreting model predictions. In: Guyon I, Luxburg UV, Bengio S et al. (eds), *Advances in Neural Information Processing Systems 30*, Long Beach, California, USA. Curran Associates, Inc., 2017, 4765–74.

[vbae049-B11] Molnar C , Casalicchio G, Bischl B. iml: an R package for interpretable machine learning. J Open Source Softw 2018;3:786.

[vbae049-B12] Obermeyer Z , Powers B, Vogeli C et al Dissecting racial bias in an algorithm used to manage the health of populations. Science 2019;366:447–53. 31649194 10.1126/science.aax2342

[vbae049-B13] Olsen LR , Zachariae HB. cvms: Cross-Validation for Model Selection. R package version 1.3.4. 2022. https://CRAN.R-project.org/package=cvms.

[vbae049-B14] Saarela M , Jauhiainen S. Comparison of feature importance measures as explanations for classification models. SN Appl Sci 2021;3:272.

[vbae049-B15] Suresh H , Guttag J. A framework for understanding sources of harm throughout the machine learning life cycle. In: *Proceedings of the 1st ACM Conference on Equity and Access in Algorithms, Mechanisms, and Optimization (EAAMO '21),* New York, NY. ACM, 2021, 1–9.

[vbae049-B16] Vickers AJ , Elkin EB. Decision curve analysis: a novel method for evaluating prediction models. Med Decis Mak 2006;26:565–74. 10.1177/0272989X06295361 PMC2577036 17099194

[vbae049-B17] Zargari Marandi R , Leung P, Sigera C et al Development of a machine learning model for early prediction of plasma leakage in suspected dengue patients. PLoS Negl Trop Dis 2023;17:e0010758. 36913411 10.1371/journal.pntd.0010758 PMC10035900

[vbae049-B18] Zucco AG , Agius R, Svanberg R et al Personalized survival probabilities for SARS-CoV-2 positive patients by explainable machine learning. Sci Rep 2022;12:13879. 35974050 10.1038/s41598-022-17953-y PMC9380679

